# Supplementary material for: Simulated forward and backward self motion, based on realistic parameters, causes motion induced blindness
Source: Sci Rep. 2017 Aug 29;7:9767. doi: 10.1038/s41598-017-09424-6 (PMC5574926; doi:10.1038/s41598-017-09424-6)
Supplement: Supplementary file 1 — Video Legend [file 41598_2017_9424_MOESM1_ESM.pdf]

Simulated forward and backward self motion, based on realistic parameters, causes motion induced blindness

Victoria Thomas, Matthew Davidson, Parisa Zakavi, Naotsugu Tsuchiya, and Jeroen van Boxtel

Supplementary Video S1 Legend:

- ‘Experimental display for Experiments 1, 2 and 3.’

Supplementary Video S2 Legend:

- ‘Experiment 4 stable mask speed display.’
